# Antibody against recombinant heat labile enterotoxin B subunit (rLTB) could block LT binding to ganglioside M1 receptor

**Published:** 2010-09

**Authors:** J Salimian, AH Salmanian, R Khalesi, M Mohseni, SM Moazzeni

**Affiliations:** 1 Department of Immunology, Faculty of Medical Sciences, Tarbiat Modares University; 2 Department of Plant Biotechnology, National Institute of Genetic Engineering and Biotechnology (NIGEB), Tehran, Iran

**Keywords:** Enterotoxigenic *Escherichia coli* (ETEC), Heat Labile Enterotoxin B Subunit (LTB), Recombinant Vaccine, GM1 Receptor Assay

## Abstract

**Objectives:**

Enterotoxigenic *Escherichia coli* (ETEC) is one of the most common agents of diarrhea among other bacterial agents. Designing and producing vaccine against these bacteria is one of the major purposes of World Health Organization (WHO). Due to presence of diverse clones of ETEC strains in the world, the use of global vaccines for ETEC infection is controversial. B subunit of heat labile toxin (LTB) was introduced as a vaccine candidate molecule by several investigators. The expression of LTB gene isolated from a local bacterial strain and investigation of its immunological property was the objective of this study.

**Materials and Methods:**

LTB gene was isolated from a local isolated ETEC, cloned and expressed using pET28a expression vector. For LTB gene expression, the three main expression parameters (IPTG concentration, time and temperature of induction) were investigated. The recombinant protein was purified (>95%) with Ni-NTA column using 6XHis-tag and used as an antigen in ELISA test.

**Results:**

The immunological analyses showed production of high titer of specific antibody in immunized mice. Anti LTB Antibody could bind to whole toxin and neutralize the toxin through inhibition of its binding to the Ganglioside M1 receptor.

**Conclusion:**

The recombinant LTB protein is a highly immunogenic molecule. Considering the LTB role in ETEC pathogenesis, it can be taken into account as one of the most important components of vaccines against local ETEC.

## INTRODUCTION

Annually, about 3 million people die due to gastroenteritis disease in the world. Viruses are the most common causes of gastroenteritis in developed countries, while bacterial agents are more common in developing countries (1–3). Enterotoxigenic *Escherichia coli* (ETEC) is the most important bacterial agent of diarrhea. Annually, ETEC strains cause 280-400 millions infected cases in children under 5 years old in developing countries and impose 400-800 thousands death per year (4). In addition to children and adults living in developing countries, disease is common among the peoples who travel to endemic: travelers disease (5, 6). The ETEC infection is also frequent after natural disasters such as earthquakes and flooding (7).

A vaccine to control ETEC infection could have a significant impact on morbidity and mortality caused by this bacterium. There are some evidences that protective immunity may be effective against this disease. Adults in developing countries are less frequently affected by disease and some travelers to endemic area are not influenced by disease even in case of having long term habitation. Therefore, designing and producing vaccine against ETEC disease is amongst the important aims of hygienic organizations such as world health organization (WHO) (8, 9).

Vaccine candidate molecule(s) have to be safe and immunogenic and induce protective immunity against broad spectrum of ETEC strains. Owing to this fact, heat labile enterotoxin (LT) B subunit is considered as a vaccine candidate molecule because: a) it is nontoxic subunit of LT molecule that play an important role in ETEC virulence and pathogenesis, b) most clinical ETEC isolate can produce LT and c) LTB is a potent immunogen and possess adjuvant properties (10, 11).

Heat labile enterotoxin consists of two different subunits; heavy (LTA) and light chain (LTB). LTA is the toxic component because of its ADP-ribosyl transferase activity that induces diarrhea via activation of adenylate cyclase which increases cyclic AMP in the intestinal cells and causes the dehydratation. LTB is a 55 KD, homopentamer of 11.6 KD peptides which join non-covalently to A subunit and binds to ganglioside GM1 receptors on the surface of enterocytes (12).

LTB has much similarity to cholera toxin B subunit (CTXB) structurally, functionally and genetically (11). Cholera vaccine (Dukoral*©*) which has license of use in 15 different countries, also contains recombinant cholera toxin B subunit (CTXB). The most important disadvantage of this vaccine is its short period of protection (3-6 month) (8, 9). The effectiveness of global vaccine against ETEC is challenged by several studies due to diverse clones of ETEC strains in the world depending on factors such as natural climates, nutrition and hygienic culture. So, specific vaccine was suggested for different geographical regions of the world (13).

In this study we describe isolation and expression of LTB gene from a local isolate of ETEC. The purification of recombinant LTB, its immunogenicity and effectiveness of antibody against rLTB in inhibition of LT binding to its receptor (GM1) is investigated as well.

## MATERIALS AND METHODS

**Bacterial strain and genomic DNA extraction**. An ETEC bacterium isolate (Reference laboratory of Bu Ali hospital, Tehran, Iran) was cultivated in LB broth. Bacterial genome was extracted by CTAB-NaCl method and its concentration was measured by spectrophotometer (OD 260 and 280nm). Electrophoresis on 1% agarose gel was also done to determine the quality of isolated DNA (14).

**Primer design and PCR**. Primer pair was designed based on deposited data in gene bank (Accession No M17874) for conserved domain of B subunit of heat labile toxin (LTB). Forward (5'TGTGCAGAATTCGCTCCTCAGTC) and reverse (5'TTACAAGCTTCTAGTTTCCATACTGATTG) primers containing *Eco*RI and *Hind*III sites (under lined) respectively for subsequent cloning procedure.

The LTB gene (312bp) was amplified using *pfu* DNA polymerase (Fermentas, Lithuania) in a reaction mixture (25 µl) containing DNA (50 ng) in the presence of 2.5 mM magnesium sulfate, 0.4 mM of each nucleotide and 0.8 pM of each primer. Cycling conditions contain initial denaturation ( 94°C, 5 min) followed by 30 cycles of 94°C for 30 sec, 58°C for 30 sec, 72°C for 60 sec and final extension at 72°C for 10 min. PCR products were analyzed by electrophoresis on 1% agarose gels and ethidium bromide staining.

**PCR product analysis and cloning**. The authenticity of PCR product was determined by restriction enzyme digestion and sequencing. The LTB gene was digested with *Eco*RI/*Hin*dIII restriction enzyme and cloned in the same site in pET28a expression vector (Novagen, Canada) under the control of chemically inducible T7 promoter. The accuracy of cloning was determined by PCR with specific primers and restriction enzyme digestion.

**Optimization of rLTB expression in *E. coli* host.** Recombinant pET28a-LTB plasmid was transformed into competent *E. coli* strain BL21DE3plysS (Stratagen, USA) by standard procedure. The rLTB expression was optimized for inducer (isopropyl-b-D-thiogalactopyranoside, IPTG) concentration (0.25, 0.5, 0.75, 1 and 1.5 mM), incubation times (1, 2, 3, 4 and 5 hours) and incubation temperatures (25, 35 and 37°C).

**Analysis of rLTB by SDS-PAGE.** Protein expression in induced *E. coli* cell was analyzed on 12% denaturized polyacrylamide gel electrophoresis. Briefly, 5 ml of the induced cells were centrifuged (5000 g, 10 min) and sonicated on ice (45s, in 75% maximum outputs). The protein concentration was determined by Bradford assay (15). Equal amounts of each sample were analyzed by SDS-PAGE. To determine the form of expressed protein (soluble vs. inclusion body), bacterial cells were centrifuged (5000 g, 10 min) and one part was resuspended in buffer A (PBS pH 7.2) and sonicated as above. Another part was resuspended in buffer B (PBS containing 8M urea) followed by incubation at room temperature for 45 min and centrifuged (5000 g, 10 min). Supernatant of two parts were collected after centrifugation and analyzed on SDS-PAGE (12%) (16).

**rLTB purification by Ni-NTA affinity chromatography column**. For purification of the rLTB, the recombinant *E. coli* cells were grown under optimized condition and resuspended in B buffer (above) and sonicated. The supernatant was separated by centrifugation (5000 g, 4° C, and 10 min) and loaded on Ni-NTA column (Qiagen, Canada). The purification was performed according to the protocol provided by manufacturer. LTB fraction was dialyzed overnight against PBS pH 7.2 and stored at-70°C until use.

**Immunoblot analysis**. As described previously, LTB is structurally very similar to B subunit of cholera toxin (CTXB). Therefore the rabbit antisera against CTXB (Sigma) produced in our laboratory by immunization of rabbit with CTXB was used for detection of LTB protein. Briefly, the separated recombinant protein bands through 12% SDS-PAGE were transferred to nitrocellulose membrane (Roche, Germany) using a Mini Trans Blot electrophoretic transfer cell (Bio-Rad, USA). The transfer process carried out using 300 mA current for 1 h in the transfer buffer (50 mM Tris, 40 mM glycine, 0.04% SDS, 20% methanol, pH 8.3). Non-specific antibody reactions were blocked by incubation of membranes in 10 ml of 5% skim milk in PBST buffer (PBS, 0.05% Tween- 20, pH 7.2) at room temperature for two hours. The membrane was then incubated for 1 h at room temperature in 10 ml of 1: 200 dilution of rabbit anti-CTXB antiserum. The membrane was washed four times in PBST buffer followed by incubation for 1 h at room temperature in 1: 2000 dilutions of anti-rabbit IgG conjugated with horseradish peroxidase (Dako, Denmark). After washing, color was developed with 3, 3′-Diaminobenzidine (DAB) (Sigma, Germany) in buffer (10 mM Tris, pH 7.5, 10 µl H_2_O_2_).

**Heat labile toxin production and purification**. For GM1 inhibition assay, it was necessary to use heat labile toxin (LT) as a positive control. Therefore, the LT was purified from an enterotoxigenic *E.coli* strain as described by Kunkel and Robertson, 1979 and Menezes *et al.*, 2006 (17, 18). Briefly, the LTB producing strains were cultivated in media containing 2% casamino acids, 0.15% yeast extract and 1% glucose at 37°C, 200 rpm for 18h. The well grown bacterial culture was incubated with polymyxin B (1mg/ml, final concentration) for further 30 minutes and centrifuged (1600 x g /20 min). Solid ammonium sulphate was added to the supernatants to 90% saturation with stirring at 4 °C and kept overnight. After centrifugation, the pellet was resolved in Tris buffer (0.02 M pH 8) and dialyzed against the same buffer at 4 °C overnight.

**GM1-ganglioside binding assay for LT**. The binding of LT to GM1-ganglioside receptor was determined by GM1-ELISA as described by Svenerholm and Wiklund, 1983 and Ma *et al.*, 2006 (19, 20). ELISA plates (NUNC, Denmark) were coated with 100µl (3.0 µg) per well of monosialoganglioside GM1 (Sigma, G-7641), dissolved in bicarbonate buffer and incubated at 37°C (2 h). BSA was used as negative control. The wells were washed (four times) with PBST and blocked by skim milk solution 2% (w/v) in PBST. The wells were loaded with serially diluted LT (100 µl per well) and then covered with 1:500 dilution of rabbit anti-CTXB primary antibody (100 µl). The wells were then incubated with 1:5000 dilution of goat anti-rabbit IgG conjugated with horseradish peroxidase (Sigma) in PBST. Each step completed with 1h incubation at 37°C and 4 times washing with PBST. Finally plates developed with 100 µL per well of OPD (Sigma) substrates in citrate-phosphate buffer (pH 5) for 20 min at room temperature in dark. After stopping the reaction with sulfuric acid (2.5 M), the plate was read at 492 nm with an ELISA reader (Dynex, USA).

**Humoral immune responses after immunization with rLTB**. Affinity purified recombinant LTB protein (20 µg) was injected subcutaneously (S.C.) along with complete Ferund's adjuvant. Up to 3 booster doses were also injected S.C. with incomplete Ferund's adjuvant with 2 weeks intervals. Bleeding was done two weeks after final booster and mouse serum were collected. Anti-LTB IgG purified by protein G on Sepharose 4B fast flow column (Sigma).

Indirect ELISA was used for antibody titration. A 96-well ELISA plates (NUNC, Denmark) were coated with LTB protein (10 µg/ml) in carbonate-bicarbonate buffer by 2h incubation at 37°C. The plates were washed with PBST (4X), blocked with skim milk solution 2% (w/v) in PBST and then incubated with a serial dilution (1: 2000 to 1/64000) of mouse anti-LTB antiserums in PBST for 1 h at 37°C. The remaining steps were performed as described above with the exception of using HRP conjugated anti-mouse antibody (Sigma) as secondary antibody. The sera collected from mice before immunization was used as negative control.

**Toxin neutralization assay**. To study the efficiency of sera from immunized mice to block the binding of LT to its receptor (GM1), a GM1 binding inhibition assay was carried out. Serially diluted sera from immunized mice were incubated (37°C, 1 h) with LT (2 µg/ 100 µl). The mixture of sera and LT was then added to the GM1 pre-coated wells and the GM1-ELISA was performed as described previously (21).

## RESULTS

**Fig. 1:**
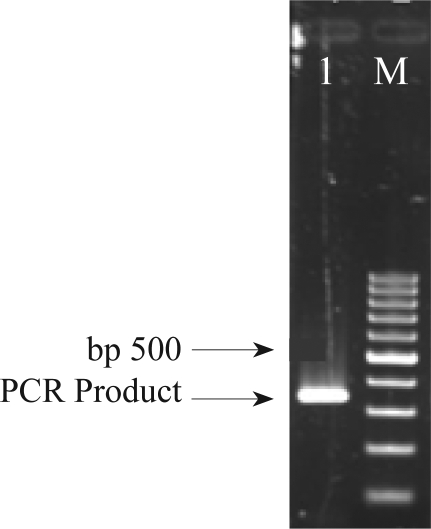
Analysis of PCR product on 1% agarose gel. Genomic DNA was extracted from ETEC and the LTB gene was amplified using the specific primers. Lane 1: PCR product of LTB gene (312 bp). M: 100 bp DNA ladder.

**LTB gene amplification and cloning**. The LTB gene from ETEC bacteria was amplified by PCR (Fig. 1). The PCR product was further analyzed by restriction enzyme digestion and sequencing in both direction (data not shown). The PCR product was digested with *Hin*dIII and *Eco*RI and cloned into pET-28a which was digested with the same restriction enzymes. Recombinant pET28a-LTB were further analyzed by sequencing and restriction enzyme analysis (data not shown).

**rLTB expression and purification**. The recombinant *E. coli* bacteria were induced in optimized condition (1 mM IPTG after 3h at 37°C). The SDS-PAG analysis show successful expression of rLTB protein (Fig. 2a). The majority of recombinant LTB was accumulated in the form of inclusion bodies (Fig. 2b). Following Ni-NTA affinity chromatography and several washing steps, the rLTB was purified from the column by elution buffer (Fig. 2c). For confirmation of purified rLTB, it was electrotranfered to nitrocellulose membrane and identified by immunoblotting. The reaction with anti CTXB antiserum (Fig. 2d) stained a protein band around 15.5 KD and reconfirmed the purification of rLTB.

**Fig. 2a:**
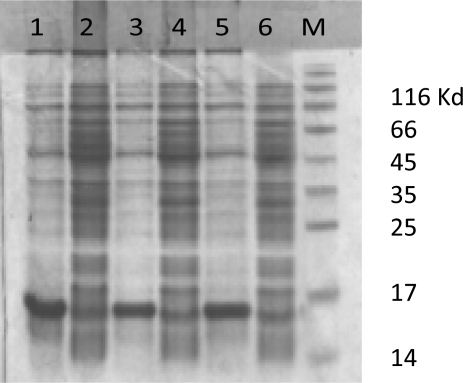
Analysis of the recombinant LTB expression after IPTG induction by SDS–PAGE. The different clones were analyzed after IPTG induction for the expression of LTB by SDS-PAGE. Lane 1, 3, 5: The induced bacteria. Lane 2, 4, 6: The same bacteria before induction. M: protein Molecular weight marker.

**Fig. 2b:**
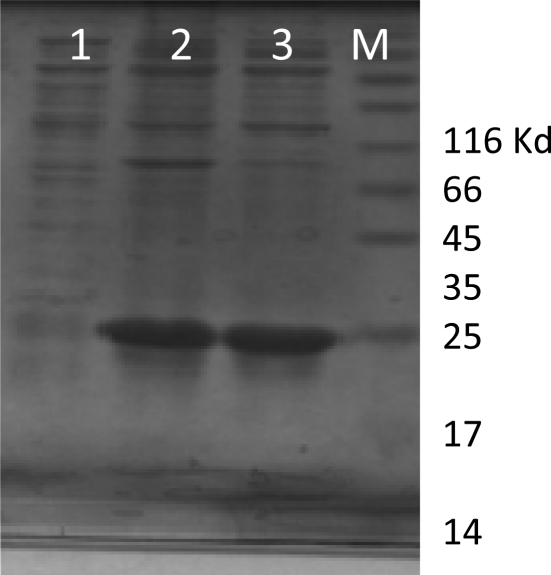
Analyses of expressed LTB: soluble protein vs. inclusion body. After induction, the cells were sonicated in the presence or absence of 8M urea and analyzed by SDS-PAGE. Lane 1: The cells extract in non denaturing condition (PBS buffer). Lane 2, 3: The cells extract in denaturing condition (PBS containing 8 M urea). M: protein molecular weight marker.

**Fig. 2c:**
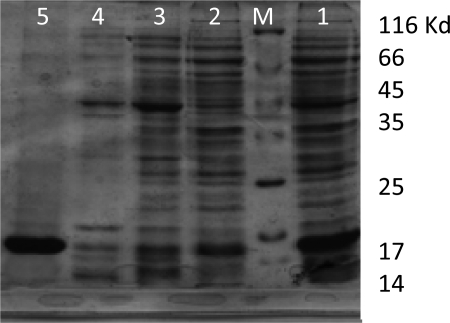
Purification of rLTB by Ni-NTA column. Lane 1: Protein sample before purification. Lane 2-4: Protein sample in washing buffer fractions before elution. Lane 5: Purified rLTB in elution buffer. M: Protein molecular weight marker.

**Fig. 2d:**
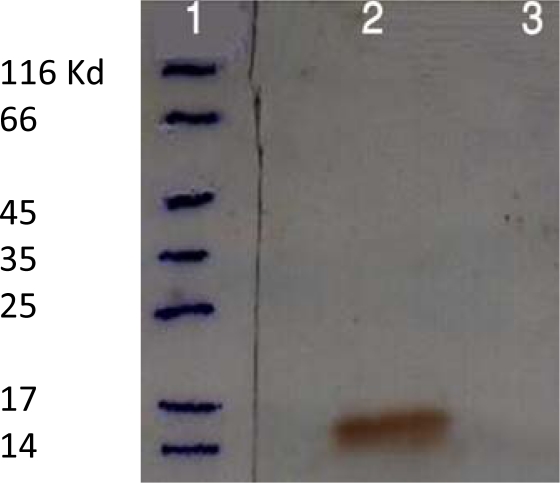
Immunoblot analyses of recombinant LTB by specific antibody. Lane 1: Protein molecular weight marker. Lane 2: Immunoblotting with anti-CTXB antibody to detect rLTB protein. Lane 3: Negative control (bacterial lysate without IPTG induction)

**Fig. 3:**
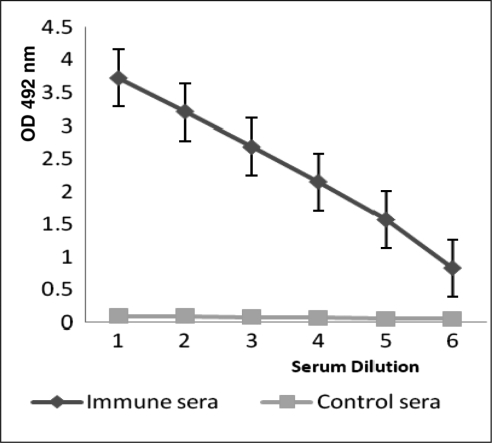
Titration of anti-rLTB antisera. Titers of IgG against rLTB were determined by ELISA in the sera of immunized mice. The sera were serially diluted (1: 2000 to 1: 64000) and used in ELISA test. The data presented as three independent measurements.

**Immune sera antibody titration**. The humoral immune responses of immunized mice were measured by ELISA technique. The data showed that high titer of anti-rLTB antibody was produced by immunized mice. As shown in Fig. 3, after one injection and 3 boosters, all mice were hyper immunized and anti-rLTB antibody titer received up to 1/64000.

**Fig. 4:**
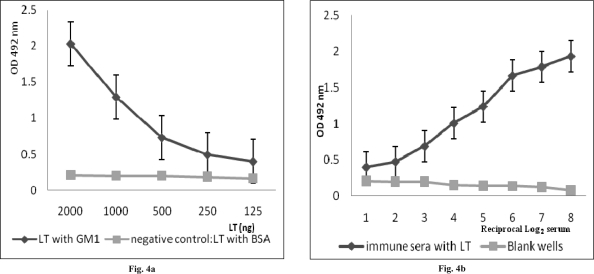
Heat Labial-toxin neutralization assay. The inhibition of the binding of LT to GM1 receptor was determined using GM1 ELISA assay. 4a: Serially diluted LT (2000-125 ng) in PBS was added to GM1 coated ELISA plates. 4b: as figure shows the serially diluted sera from immunized mice could block the binding of LT to GM1 receptor. Each graph shows the mean OD±SD in three independent experiments.

**Analysis of neutralizing antibody**. To examine the biological activity of rLTB-specific antibodies, their efficiency to neutralize the heat-labile toxin was investigated by a GM1-binding inhibition assay. The GM1-ganglioside binding assay of LT demonstrated the specific binding of purified LT to GM1. The titration curve shows that purified LT binds to GM1 receptor from 2 µg to 125 ng (Fig. 4a). For analysis of neutralizing antibody, serum samples from immunized mice were subjected to GM1-ELISA. Serum antibodies from control mice were used as negative controls. Binding of LT to the coated GM1 ganglioside was blocked (up to 80%) by serum samples isolated from immunized mice. The sera from control mice or PBS have shown no reaction (Fig. 4b).

## DISCUSSION

It has been estimated that 30-70% of gastroenteritis is caused by bacteria. Among bacterial agents, enterotoxigenic *E. coli* (ETEC) is the most common agent. Nowadays, ETEC morbidity and mortality rate has been increasing due to emergence of ETEC antibiotic resistance and inefficient treatment. Developing countries including Iran with tropical weather are among the regions that encounter sanitary issues such as ETEC diarrhea especially in children under 5 years age (1–3). Taking these issues in to consideration, trying to preclude ETEC infection is one of the main sanitary purposes.

Literature review indicates the attempts of several groups on ETEC vaccine development to control the disease (8, 9). One of the major points that should be considered in vaccine development is induction of long term (at least 2 years) protection in most immunized individuals. Moreover vaccine candidate molecule/s has to be selected from the conserved set of pathogenic important molecules, so that most ETEC strains posses them (8, 9). The effectiveness of global vaccine against ETEC is challenged by several studies due to the presence of diverse clones of ETEC strains in different parts of the world. So, specific vaccine was suggested for different geographical regions (13). Regarding all findings, this study was done using a regional strain of ETEC and LTB molecule as binding domain of heat labile toxin, which is reported that most of ETEC clinical isolate posses it. It was reported that the presence of antibody against LTB can block the binding of the toxin to epithelial cells and decrease the related effects of toxin release (water and electrolyte loss) (22, 23). Yamamoto and coworker was cloned the LTB and reported its necessity for developing vaccine against ETEC (24). Immunomodulatory effect was also reported for the LTB (25). Based on this property, it shows adjuvant effect and up-regulates the immune response against the vaccine components (26).

In this study, the gene encoding for LTB was isolated from local isolate of enterotoxigenic *E. coli* that causes diarrhea in humans. A DNA fragment of the expected size ( near 300 bp) was amplified by PCR and expressed using chemically inducible T7 promoter (pET28a).

The best condition for the recombinant rLTB expression was achieved by changing the IPTG concentration, bacterial culture temperature and duration of induction. The expression yield increased with ascending concentration of IPTG from 0.25 to 1 mM, while increasing inducer concentration to 1.5 mM showed no effect on protein expression. Therefore it can be postulated that in this situation the T7 promoters were saturated in 1 mM IPTG. The require time after induction was another factor that has been investigated. Protein expression increased with bacterial culture duration up to 3 hours. But after 4 and 5 hours, expressed protein decreased slightly. It seems that in long term induction, following high level expression of rLTB, some of the cells don't tolerate this condition and lysed. In this case, the recombinant protein from lysed cells releases into culture media and degraded. In order to inhibit protein accumulation in inclusion body and increasing its solubility, culture incubation was performed at room temperature (25°C). In this temperature bacterial cell metabolism and protein synthesis decrease but, rLTB showed the tendency to aggregate, yet. However, high expression of rLTB was an advantage but its accumulation in the form of inclusion bodies makes limited complication in protein extraction process. De Mattos Areas AP, was reported the same problem for CTB expression (27). Rezaee *et al.* used *Saccharomyces cerevisiae* for expression of LTB and reported that it could be an effective strategy to protect the animal model against ETEC infection (28).

The rLTB was separated on SDS–PAGE and its biochemical nature was confirmed by immunoblotting using anti-CTXB antiserum. Based on 6XHis-tag and using Ni-NTA column, the recombinant protein became highly purified (> 95%). The purified rLTB was used for immunization of mice. The ELISA test results showed that all immunized mice produced high titer of anti LTB antibody after four times injection with rLTB. This result can be related to LTB function as immunostimulator and its adjuvant property (26). GM1 ganglioside binding assay is widely used for detection of LT and cholera toxin (19–21). This study show that antibody against rLTB could neutralize the toxin and efficiently inhibited it's binding to the corresponding receptor (GM1). This data demonstrate that antibody against recombinant LTB can recognize native form of LTB in holotoxin and possess the ability to block the LT binding to its receptors.
